# NanoDSF Screening for Anti-tubulin Agents Uncovers New Structure–Activity Insights

**DOI:** 10.1021/acs.jmedchem.5c01008

**Published:** 2025-08-15

**Authors:** Viktoriia Baksheeva, Romain La Rocca, Diane Allegro, Carine Derviaux, Eddy Pasquier, Philippe Roche, Xavier Morelli, François Devred, Andrey V. Golovin, Philipp O. Tsvetkov

**Affiliations:** † CNRS, INP, Inst Neurophysiopathol, Aix-Marseille Univ, 13005 Marseille, France; ‡ CNRS, INSERM, Institut Paoli Calmettes, CRCM, Centre de Recherche en Cancérologie de Marseille, Aix-Marseille Univ, 13009 Marseille, France; § PINT, Plateforme Interactions moléculaires Timone, Faculté des Sciences Médicales et Paramédicales, 128791 Aix-Marseille Univ, 13005 Marseille, France; ∥ Faculty of Bioengineering and Bioinformatics, Belozersky Institute of Physico-Chemical Biology, Lomonosov Moscow State University, 119991 Moscow, Russia; ▽ Department of Computational Biology, Sirius University of Science and Technology, 354340 Sirius, Russia

## Abstract

Microtubule targeting agents (MTAs) constitute a vital category of tubulin-binding compounds deployed across anticancer therapies. Despite the array of MTA drugs developed by pharmaceutical entities, the quest for novel efficacious molecules continues unabated. We unveil an innovative in vitro MTA screening methodology employing nano-differential scanning fluorimetry (nanoDSF), presenting distinct advantages over known assays. This novel approach not only assesses compound-tubulin binding but also quantitatively analyzes its impact on tubulin polymerization, facilitating structure–activity relationship discovery. The proposed nanoDSF assay was rigorously validated using the Prestwick Chemical Library, which encompasses 1520 approved compounds, successfully identifying all previously known MTAs. This screening has unearthed potential antitubulin agents among drugs currently utilized for unrelated medical conditions, offering insights into their mechanisms of action in inhibiting cancer cell proliferation and/or inducing cytotoxicity. Finally, we have identified a previously unrecognized structure–activity relationship within the carbendazim and phenothiazine drug clusters, providing valuable insights for the rational optimization of compounds from these families. These discoveries open new opportunities for drug repositioning of the newly identified MTAs and significantly streamline the screening process of large chemical libraries for MTAs with novel chemical scaffolds.

## Graphical abstract

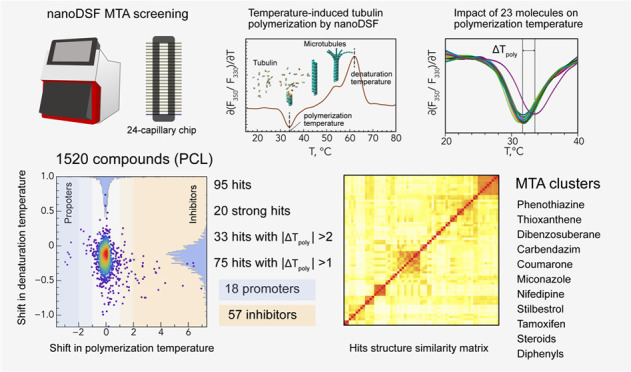

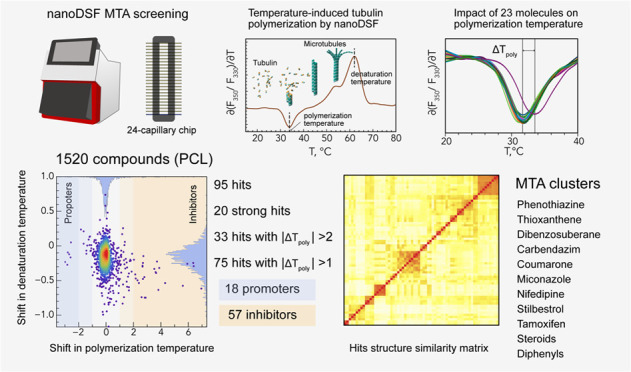

## Introduction

Microtubules (MT), polymeric structures composed of tubulin heterodimers, possess the dynamic ability to elongate or shorten (Figure 1A) in response to environmental shifts. The dynamic behavior of MTs is essential for cell motility and division, playing key roles in cytoskeletal rearrangement during cell migration and the accurate positioning of chromosomes during mitosis. This necessitates a sophisticated level of control over the MT dynamics. To achieve such precise regulation, cells have developed a complex network of microtubule-associated proteins (MAPs) that meticulously orchestrate the dynamics of MTs.^1^ Any disruption in this fine-tuned process can adversely affect cell migration and block mitosis and thus potentially be fatal for the cell. This explains why MT dynamics has become the focus of a wide range of pharmacological agents.

**Figure 1:**
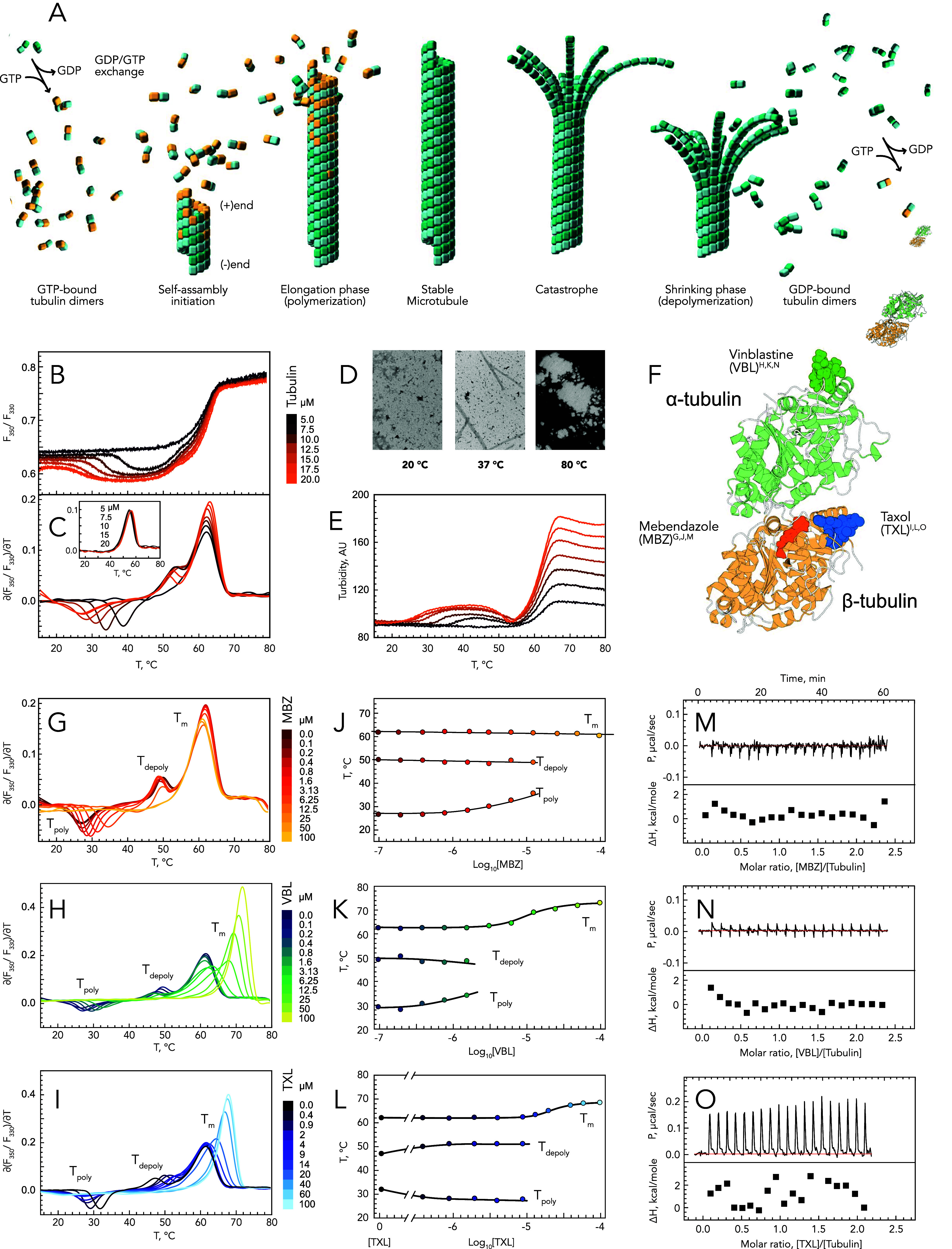
Monitoring tubulin polymerization with nano-differential scanning fluorimetry (nanoDSF). (A) Different stages of tubulin polymerization in MTs. (B,C) Polymerization and denaturation of tubulin at different tubulin concentrations followed by nanoDSF; the inset represents tubulin denaturation followed by nanoDSF in the nonpolymerizing buffer. (D) Transmission electron microscopy (TEM) of tubulin at different temperatures. (E) Polymerization and denaturation of tubulin at different tubulin concentrations followed by turbidimetry. (F) Structures of mebendazole (MBZ), vinblastine (VBL), and taxol (TXL) complex with tubulin heterodimer visualized from structures with PDB IDs: 5J2T, 7OGN, and 8UTN. (G–I) Polymerization and denaturation of tubulin in the presence of different concentrations of MBZ, VBL, and TXL, respectively, followed by nanoDSF. (J–L) Dependence of the temperatures of polymerization (*T*_poly_), depolymerization (*T*_depoly_), and denaturation (*T*_m_) of tubulin from the concentration of MBZ, VBL, and TXL, respectively. (M–O) Isothermal titration calorimetry (ITC) curves of tubulin titration by MBZ, VBL, and TXL, respectively, in the polymerization buffer.

Microtubule targeting agents (MTAs) are a class of tubulin-binding compounds that are used not only for cancer treatment but also as anthelmintic, antibacterial, and antifungal drugs. Moreover, recently, MTA administration was proposed as a new strategy for the therapy of neurodegenerative diseases.^2,3^ The MTA interaction with tubulin significantly impacts MT dynamics, thus perturbing vital cellular processes. MTAs differ by their binding sites on tubulin^4,5^ (Figure 1F) and are grouped in two classes by their ability to induce or inhibit MT formation. The first MTAs with anticancer activity were originally obtained from plants^6^ and were subsequently subjected to chemical modifications to yield new compounds with superior anticancer properties. While pharmaceutical companies have developed various MTA anticancer drugs, the demand for new molecules remains high, driving researchers to explore additional natural sources of MTAs.^7^ These new compounds should exhibit increased specificity and broader efficacy across different tumor types and should be capable of overcoming drug resistance observed in certain tumors, which may arise from MTA interplay with MAPs.^8,9^ Structure-based rational design of MTAs continues to play a crucial role in drug discovery.^10,11^ However, small chemical modifications of existing drugs often fail to produce MTAs with significantly different characteristics due to their shared scaffolds. Therefore, identifying MTAs with potentially superior anticancer properties requires screening large and structurally diverse libraries of chemical compounds.

There are several molecular screening assays based on different biophysical methods that allow for identification of the interaction between a target protein and potential binders. Among them, the thermal shift assay (TSA) has become widely used for early stage drug discovery in the last years because it is accessible (RT-PCR equipment is sufficient) and is high throughput (adapted for 96, 384, and 1536-well plates) with a low material consumption.^12^ This technique facilitates the assessment of protein thermostability (melting or denaturation temperature, *T*_m_) in the presence of screening compounds, thereby providing evidence of an interaction. However, like many molecular assays, it generates a certain number of false positive and false negative results. This phenomenon arises because, first, not all interactions between the protein and compounds lead to a significant change in protein thermostability, as detected by TSA, and, second, not every interaction impacts the biological process regulated by the protein, which is the intended target of new drug therapies. This is especially relevant for tubulin, which possesses multiple ligand-binding sites. Consequently, there is no direct correlation between the extent of tubulin stabilization by a compound and its efficacy as a polymerization inhibitor or promoter. Thus, molecular screening assays that not only demonstrate tubulin–compound interactions but also elucidate the effect of the compound on tubulin polymerization (the specific process targeted) are essential for the efficient discovery of MTAs.

To address these challenges, we introduce an *in vitro* functional MTA screening methodology utilizing nanoDSF. Unlike RT-PCR-based assays, nanoDSF does not require fluorescent dyes as it monitors the intrinsic fluorescence of proteins. Remarkably, this approach enables a dual readout, allowing simultaneous detection of both tubulin stabilization by the compound and its effect on tubulin polymerization. We applied the nanoDSF screening assay to the Prestwick Chemical Library (PCL), which comprises 1520 approved compounds. This application not only validated the assay with known MTAs contained within the library but also led to the identification of approximately 100 compounds demonstrating MTA activity.

## Results

### Monitoring Tubulin Polymerization Using Nano-Differential Scanning Fluorimetry

To monitor tubulin polymerization, we used a nanoDSF instrument from NanoTemper Technologies, which enables tracking of the protein’s intrinsic fluorescence at 330 and 350 nm across a temperature range of 15–95 °C. The ratio of these two signals (*F*_350_/*F*_330_) reflects the exposure of the protein’s tryptophan side-chain aromatic groups to the solvent, allowing for the observation of protein denaturation upon heating the sample. We have noticed that the dimerization interface of tubulin contains several tryptophan residues, potentially enabling the use of nanoDSF not only to monitor tubulin denaturation but also to observe the formation of MTs. To test this hypothesis, we subjected tubulin samples at varying concentrations to heating using nanoDSF in a polymerization buffer, wherein tubulin has the capability to polymerize upon reaching physiological temperatures (Figure 1B,C). At a low subcritical concentration of tubulin, we detected only a single transition around 62 °C, indicative of tubulin denaturation denoted further as *T*_m_. Consistently, at higher concentrations of tubulin, two additional transitions emerged on the thermogram. The first transition, occurring between 26 and 39 °C (depending on tubulin concentration), displayed a signal opposite that of tubulin denaturation, suggesting that tryptophan side-chain aromatic groups were concealed from the solvent rather than exposed. This event most probably corresponds to tubulin polymerization. The subsequent transition, occurring between 50 and 55 °C, matched the magnitude of signal change of the first transition but was opposite in direction, strongly suggesting it correlates with the depolymerization of MTs. These transitions are also accompanied by changes in sample turbidity at 350 nm, with an increase followed by a decrease, returning to baseline around 55 °C (Figure 1E). The polymer state of tubulin under nanoDSF experimental conditions was confirmed using TEM at different temperatures (Figure 1D), thus validating nanoDSF as a tool to study the impact of compounds on tubulin polymerization. Henceforth, we denote the temperatures corresponding to the minima and maxima of the first derivative of the *F*_350_/*F*_330_ ratio as the apparent temperatures of tubulin polymerization (*T*_poly_) and depolymerization (*T*_depoly_), respectively.

Given that tubulin has multiple binding sites influencing its polymerization, we evaluated the interaction of tubulin with three MTAs—Vinblastine (VBL), Taxol (TXL), and Mebendazole (MBZ)—each binding to distinct sites (Figure 1F). This was done to assess the capability of the nanoDSF assay to detect interactions between tubulin and MTAs. Therefore, tubulin samples in the presence of increasing concentrations of MBZ, VBL, and TXL were subjected to heating from 15 to 80 °C using the nanoDSF instrument. This induced markedly distinct alterations in tubulin polymerization and denaturation profiles (Figure 1G–I). A gradual increase in MBZ concentration resulted in a notable shift of *T*_poly_ to higher temperatures without affecting *T*_depoly_ and *T*_m_ (Figure 1G,J), until MBZ reached a concentration of 100 μM. At this concentration, MBZ entirely inhibited MT formation, resulting in the disappearance of the first two transitions (Figure 1G, yellow curve).

Contrary to MBZ, VBL is able not only to inhibit tubulin polymerization in substoichiometric amounts but also to decrease the temperature of depolymerization (Figure 1H,K). VBL is known to sequester tubulin into spiral structures. Therefore, the observed changes in *T*_poly_ and *T*_depoly_ values and the shallower peak slopes may indicate structural transitions between MTs and spirals rather than dimers. This also explains the increase in the height of the denaturation peak since it would induce the exposure to the solvent of tryptophans not only from the tubulin core but also from the dimerization interface. Moreover, the marked increase in the denaturation temperature may not result directly from ligand binding but rather from mutual stabilization of tubulin dimers when arranged within spiral structures.

Unlike MBZ and VBL, TXL—known to promote MT formation—shifts *T*_poly_ and *T*_depoly_ in opposite directions (refer to Figure 1I,L). At a certain TXL concentration, the characteristic transitions become indistinguishable: *T*_poly_ drops below 15 °C, while the depolymerization transition merges with the denaturation peak. Thus, similarly to VBL, high concentrations of TXL lead to an increase in the height of the denaturation peak, likely reflecting the unfolding of tubulin that remains assembled in MTs at the onset of denaturation. Despite this similarity, the denaturation profiles of tubulin differ markedly between TXL and VBL. Specifically, a detailed examination of tubulin denaturation peaks with increasing concentrations of VBL reveals a sequence where initially, the peak’s magnitude decreases, followed by the peak becoming asymmetric, and ultimately, it increases in amplitude and shifts to higher temperatures. In contrast, TXL leads to a gradual increase in both the amplitude and *T*_m_ of tubulin’s symmetric denaturation peak. This may reflect differences in site accessibility,^5^ which in turn could influence the dynamics of compound exchange between free and bound states,^13^ thereby differentially affecting tubulin denaturation.

Ultimately, the apparent affinity constants of compounds could be independently estimated by analyzing both the *T*_m_ shift^14^ and the alterations in fluorescence signal^15^ at 15 °C, which is particularly advantageous under experimental conditions where conventional reference methods, like ITC, are ineffective (Figure 1M–O). Moreover, the nanoDSF assay is easier to set up and requires significantly less material than ITC.

### Application of Nano-Differential Scanning Fluorimetry for Microtubule Targeting Agent Screening

Thus, tracking temperature-induced tubulin polymerization with nanoDSF enables the detection of shifts in the polymerization temperature (Δ*T*_poly_) across a broad concentration range with high sensitivity. This approach facilitates the qualitative assessment of MTAs’ effects on tubulin polymerization, allowing for their comparative evaluation based on this criterion. Additionally, further heating reveals the impact of MTAs on tubulin’s thermostability (Δ*T*_m_). By measuring these two distinct parameters, the first directly related to the tubulin function targeted by MTAs and the second reflecting the structural influence of MTAs on tubulin, this approach emerges as highly promising for MTA screening. Advanced nanoDSF instruments, such as the automated Prometheus NT.Plex, are equipped to conduct high-throughput screening of chemical libraries for compounds targeting tubulin. To evaluate the efficacy of our novel MTA screening methodology, we applied it, as a proof of concept, to the PCL, which comprises 1520 approved drugs (Figure 2A–C). Since *T*_poly_ is highly sensitive to tubulin concentration (Figure 1B,C), each run included a control sample, and the polymerization temperature shifts (Δ*T*_poly_) were calculated relative to this control. This approach helped to minimize the variability of Δ*T*_poly_ values across experiments. Notably, *T*_poly_ among 67 control samples followed a normal distribution, with a standard deviation of 0.5 °C, while the standard deviation of *T*_poly_ in runs without hits containing one control and 23 molecules was twice as low, at just 0.25 °C.

**Figure 2:**
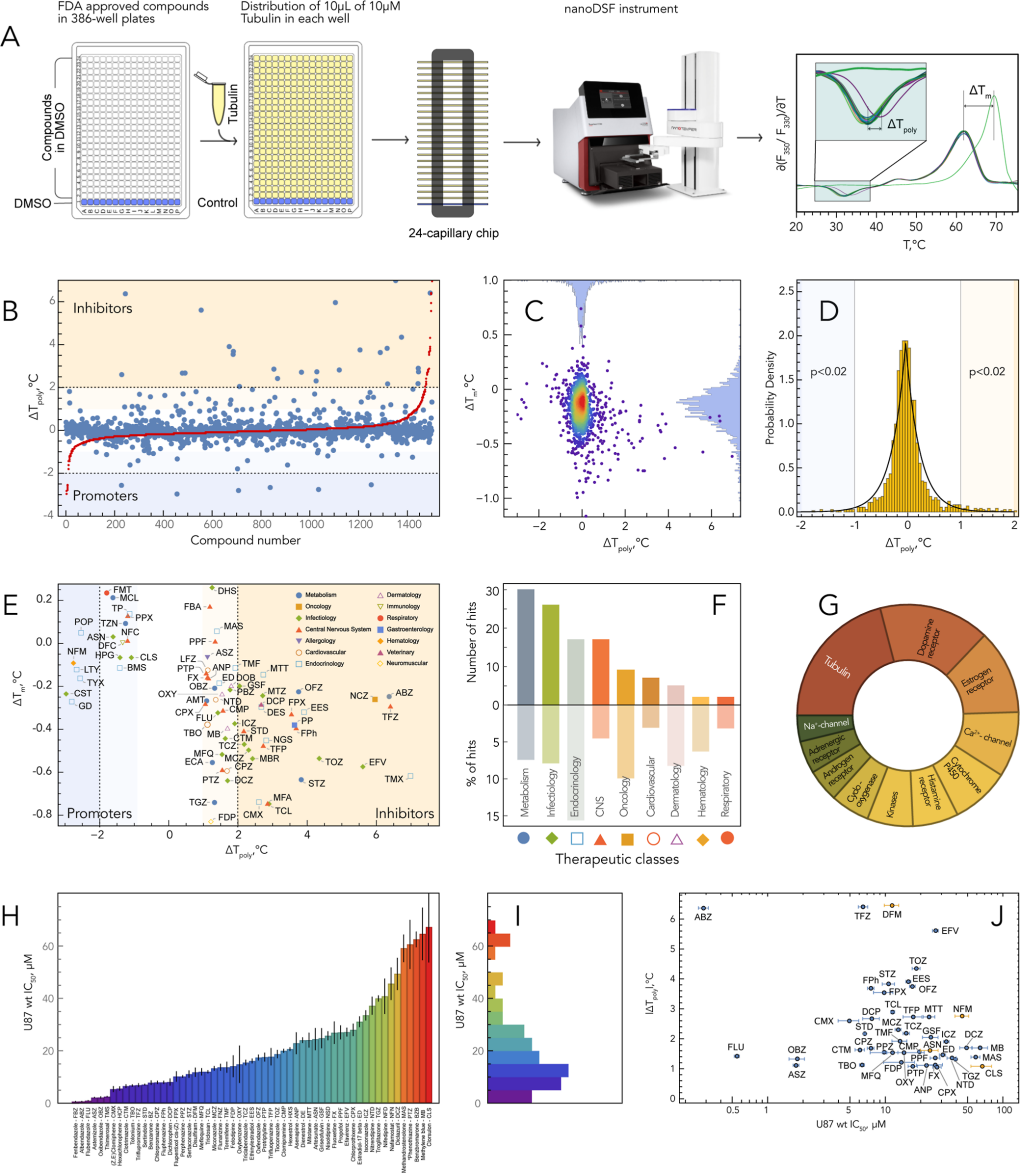
NanoDSF screening of PCL of 1520 approved compounds. (A) NanoDSF screening workflow. (B) Results of PCL screening. Red dots represent sorted data points. (C) Distributions of Δ*T*_poly_ and Δ*T*_m_ values represented as 1D histograms and 2D density plot. (D) Δ*T*_poly_ histogram and its fitting with Laplace distribution. (E) 2D distributions of Δ*T*_poly_ and Δ*T*_m_ values of hits with their therapeutic classes (some hits out of plot range, see Supporting Information tables for Δ*T*_poly_ and Δ*T*_m_ values for all hits). (F) Distribution of hits in therapeutic classes. (G) Known primary targets of hits. (H,I) IC_50_ of hits for U87MG cancer cell line and its distribution. (J) 2D distributions of absolute value of |Δ*T*_poly_| and IC_50_ of hits (orange points have negative Δ*T*_poly_).

We observed that in the presence of 20 compounds (1.3% of PCL), tubulin exhibited no polymerization, suggesting either a complete inhibition of MT formation or the initiation of MT formation at temperatures below 15 °C. These compounds are henceforth categorized as strong hits. Among these, nine are already known as MTAs (Table 1), three are suspected of having MTA activity (Auranofin (AUF), Ebselen (EBS), and Riboflavin (RBF)), and eight (Aprepitant (APT), Benzarone (BZ), Benzbromarone (BZB), Benziodarone (BZI), Bithionol (BTN), Hexachlorophene (HCP), Nifedipine (NFD), and Nisoldipine (NSD)) are newly identified as exhibiting MTA activity, previously unreported. For the remaining 1500 drugs, tubulin polymerization occurred, enabling their classification based on two metrics: Δ*T*_poly_ and Δ*T*_m_. The initial findings, along with 1D and 2D distributions of these metrics, are depicted in Figure 2B–D with standard deviation equal to 0.7 and 0.2 °C for Δ*T*_poly_ and Δ*T*_m_, respectively. Both values demonstrate Laplace distribution; compounds causing a Δ*T*_poly_ shift greater than 2 °C were designated as hits with *p* < 0.0004. Those with a 1 °C < Δ*T*_poly_ < 2 °C are also considered potential MTAs, termed weak hits (*p* < 0.02), meriting further investigation.

**Table 1:** List of Drugs from PCL with the Most Important Effect on Tubulin Polymerization

Name	Therapeutic class	Targets	Impact on MTs	Cancer treatment
**Known MTAs**				
Colchicine	metabolism	tubulin	prevents MT assembly and thereby disrupts inflammasome activation^37^	derivatives regarded as potential chemotherapy drugs^83^
Docetaxel	oncology	tubulin	disrupts normal MT dynamics and thereby stops cell division^84^	chemotherapy agent utilized in various cancers^85^
Fenbendazole	infectology metabolism	tubulin	moderate affinity to mammalian tubulin^48^	moderate antineoplastic activity^48^
Mebendazole	infectology metabolism	tubulin	selectively inhibits tubulin polymerization via interaction with colchicine-binding site of β-tubulin^86^	repositioned as a prospective anticancer agent^47^
Paclitaxel	oncology	tubulin	stabilizes the MT polymer and protects it from disassembly^87^	anticancer drug^88^
Podophyllotoxin	metabolism	tubulin	prevents polymerization of tubulin by binding to colchicine site^89^	antitumor, derivatives applied in chemotherapy^90^
Dienestrol	endocrinology	ERα, ERβ	inhibits MT assembly in vitro by binding to the site analogous to the colchicine site^26^	carcinogenic^91^
Hexestrol	endocrinology oncology	ERα, ERβ	inhibits MT assembly in vitro by binding to the site analogous to the colchicine site^26^	carcinogenic^92^
Thiomersal	infectology	InsP_3_R	inhibits tubulin polymerization in vitro^27^	induces apoptosis in some cancer cell lines^93^
**Suspected for MTA activity**				
Auranofin	metabolism	NF-κB kinase β; PRDX5	inhibits phagocytosis which could be linked to MT modulation^29^	cytotoxic to mutant p53 cancer cells^45^
Ebselen	metabolism central nervous system (CNS)	AChE, SEH	in high doses disrupts MTs in cells^32^	suppresses cancer cell growth^33^
Riboflavin	metabolism ophthalmology	FMN	rescues cytoskeletal alterations in patients with RTD^34^	potential adjuvant in chemoradiotherapy^35^
**Newly detected MTAs**				
Aprepitant	metabolism	NK 1 receptor	unknown	potential antitumor agent^40^
Benzarone	rheumatology	nonpurine XO; SLC22A12; EYA3	unknown	inhibits tumor growth in animal model^36^
Benzbromarone	cardiovascular	uric acid uptake	unknown	predicted as a therapeutic drug for lung adenocarcinoma (LUAD)^44^
Benziodarone	cardiovascular	uric acid uptake	unknown	not used
Bithionol	dermatology	ADCY1	unknown	synergistic with paclitaxel in ovarian cancer^42^
Hexachlorophene	infectology	G6PDH, SHP2	unknown	suppresses proliferation in nonsmall cell lung cancer (NSCLC) model^42^
Nifedipine	cardiovascular	cytochrome P450 3A4	unknown	reverses drug resistance of cancer cells^43^
Nisoldipine	cardiovascular	DHP channel	unknown	not used

Among both strong and weak hits, we discovered that 30 compounds are classified within the therapeutic category targeting metabolism; 26 are utilized in infectious diseases, 17 in endocrinology, and 17 in the treatment of CNS disorders (see Figure 2E,F). When examining the therapeutic effects of the drugs that influence tubulin polymerization, we identified 27 compounds with antifungal properties, 19 with antineoplastic effects, 16 with antibacterial activity, and 9 each with anti-inflammatory, antipsychotic, and anthelmintic effects. As expected, the main known target of those hits was tubulin; still, more than 75% of hits has another protein listed as the “main” target (Figure 2G). Therefore, tubulin should also be considered a significant target for these compounds, which could explain the molecular mechanism of action of some compounds, or the side effects associated with the clinical use of these molecules.

To assess whether compounds with MTA activity also demonstrate cytotoxic effects, cell survival assays were conducted on the human U87MG glioblastoma cell lines at varying concentrations of some identified compounds. Our analysis revealed that for approximately 50% of these compounds (54 molecules), the IC_50_ value was less than 40 μM, while for about 20% (19 compounds), it was under 10 μM (Figure 2H–J, Tables S1–S8). The IC_50_ values showed no significant correlation with the change in the tubulin polymerization temperature (Δ*T*_poly_) (Figure 2J).

### Structure–Activity Relationship of Some Newly Identified Microtubule Targeting Agents

Furthermore, we analyzed the structural similarity among all of the hit compounds. To achieve this, we initially computed a structure similarity matrix detailing the pairwise distances between compounds, utilizing “Morgan Connectivity” fingerprints. Subsequently, we derived a cluster hierarchy based on this matrix (Figure 3A) and reorganized the structure similarity matrix in accordance with the identified clustering, excluding compounds with minimal structural resemblance (Figure 3B, hits with small structural similarity are listed in Table S8). This process enabled us to identify and better visualize several clusters of molecules with analogous structures within the hits (Figure 3A,B). Thus, various small clusters that include both previously identified and novel MTAs are identified (Figure 3A,B,F–L, Tables S3–S7), two prominent clusters are highlighted, composed of established MT inhibitors: carbendazim (Figure 3D, Table S2) and phenothiazine (PTZ) derivatives (Figure 3E, Table S1). In the last cluster, we identified two pairs of molecules, perphenazine (PPZ) and fluphenazine (FPh), as well as chlorpromazine (CPZ) and triflupromazine (TFZ), wherein the substitution of a chlorine atom (–Cl) at the second position of the PTZ scaffold with a trifluoromethyl group (−CF_3_) (see Figure 3D) leads to a significant increase in Δ*T*_poly_. To gain deeper understanding of this phenomenon, we employed funnel metadynamics to simulate the docking of these four molecules, along with colchicine as a control, into the colchicine binding site of β-tubulin—also recognized as the binding site for PTZ derivatives.^16^ The docking of colchicine to β-tubulin resulted in a center-of-mass position that was consistent with the X-ray crystallographic data (data not shown). Next, a comparative analysis of the two pairs of compounds showed that the introduction of trifluoromethyl groups generally altered both the position and affinity of the molecules (Figure 4A,B). In the CPZ–TFZ pair, the difference was most pronounced, with the trifluoromethyl group penetrating deep into the protein cavity and “dragging” the entire molecule with it. In the PPZ–FPh pair, the trifluoromethyl group also played a key role in forming effective contacts within the hydrophobic region of the β-sheet near the colchicine binding site. According to our calculations, the affinity of FPh was significantly higher than that of PPZ. Comparison of TFZ and FPh suggests that their inhibition efficiencies arise from different mechanisms: while FPh exhibits high affinity, TFZ binding leads to substantial rearrangements in the interfacial interactions between α- and β-tubulin subunits.

**Figure 3:**
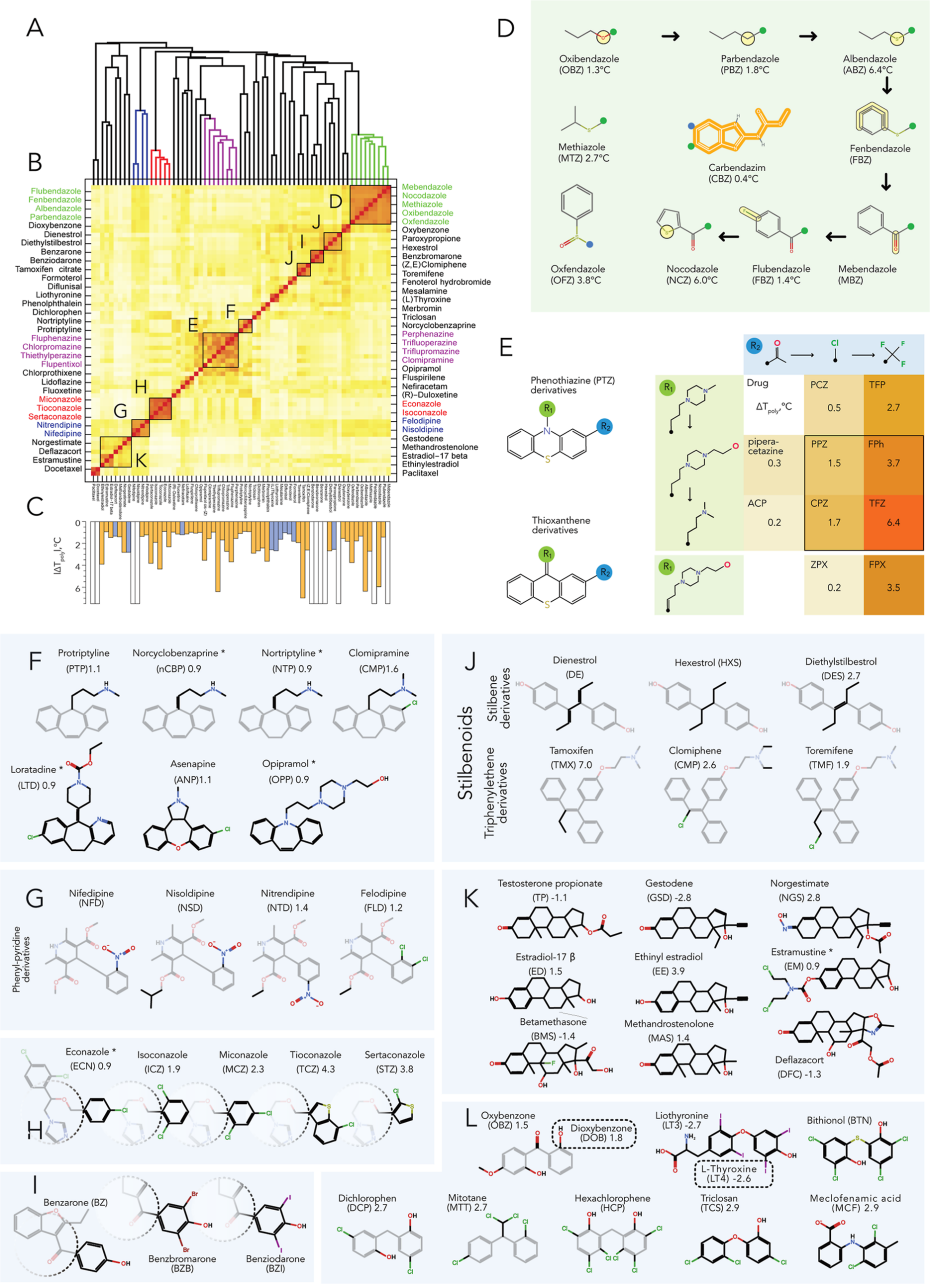
Structure–activity relationship of some hits. (A) Hierarchical dendrogram representing chemical clusters of hits. (B) Sorted chemical similarity matrix for hit compounds that have at least one similar compound among the hits. (C) Absolute value of Δ*T*_poly_ of hits: polymerization inhibitors are shown in orange and promoters in blue bars. Strong hits that completely inhibit tubulin polymerization are shown in white bars. (D) Structures and Δ*T*_poly_ of CBZ derivatives. Each next modification is highlighted in light yellow. (E) Structures and Δ*T*_poly_ of PTZ derivatives. (F–L) Tubulin inhibitors grouped by scaffold similarity, with corresponding structural formulas. Numbers indicate Δ*T*_poly_ values where applicable; * denotes compounds with low impact on tubulin polymerization (nonhits).

**Figure 4:**
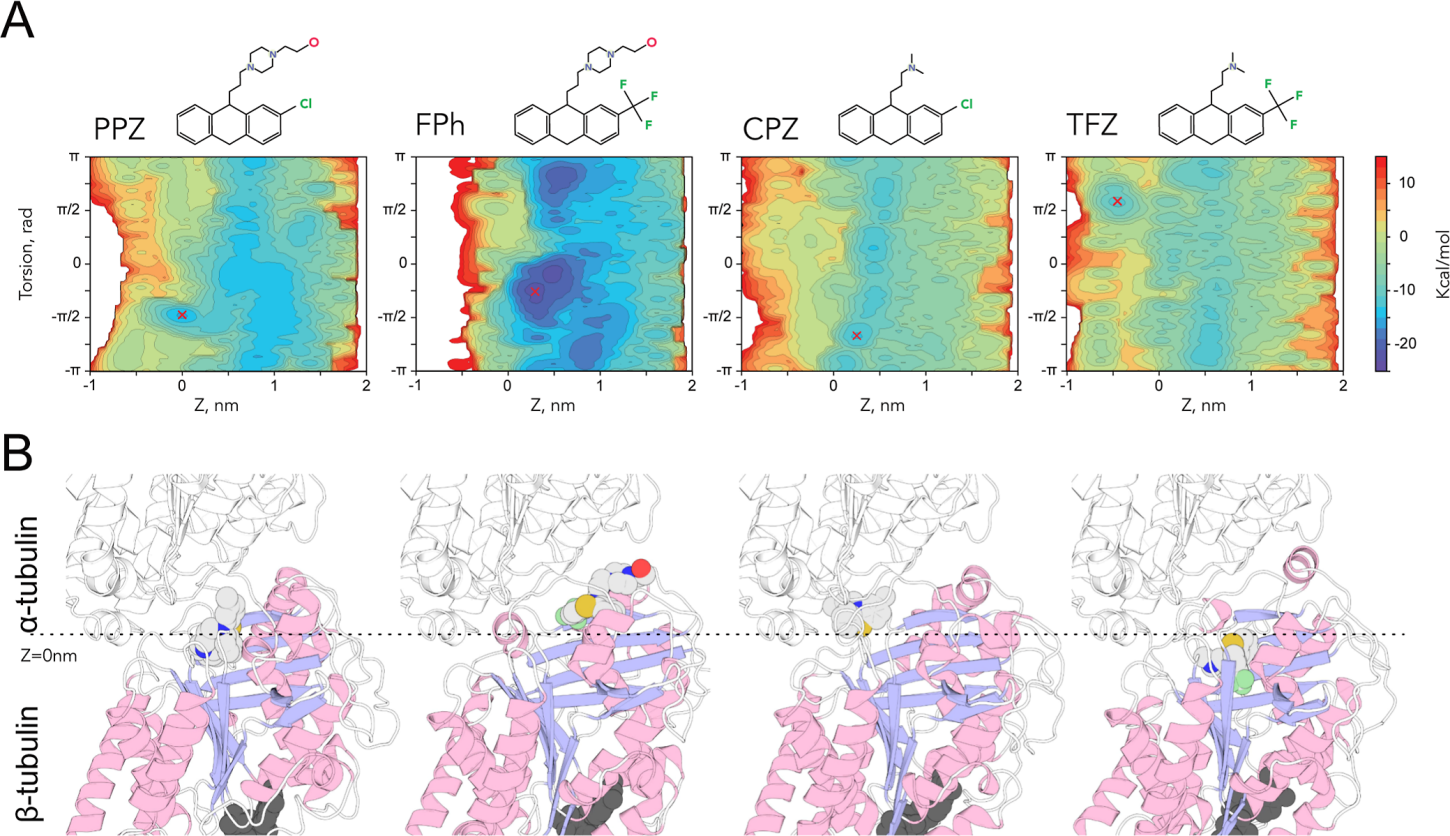
Funnel metadynamics simulation for PTZ derivatives. (A) Funnel metadynamics simulation of the interactions between compounds PPZ, FPh, CPZ, and TFZ upon binding with the β-tubulin subunit. The 2D plot represents the free energy profile of compound binding, with coordinates as follows: the *x*-axis indicates the distance from the center of mass (COM) of the CLH (as determined by X-ray data, zero value) to the COM of the compound, while the *y*-axis shows the torsion angle representing the arbitrary rotation of the compound molecule relative to tubulin within the interaction plane. Stable binding modes are marked with a red cross. (B) Visualization of the binding modes of compounds PPZ, FPh, CPZ, and TFZ with corresponding β-tubulin conformations denoted by red cross minima. The protein molecules are shown in the cartoon mode, colored in blue and light pink, while the compound molecules are displayed as spheres with carbon atoms in gray, chlorine atoms in yellow, and fluorine atoms in light green. The dashed line represents the position of the CLH COM.

## Discussion

### New Approach for Microtubule Targeting Agent Screening

MTAs are an important class of compounds widely used in the treatment of various diseases, including antifungal, antibacterial, antihelminthic, and antineoplastic therapies. Moreover, there is a growing body of evidence that MT stabilizing MTAs could be used for treatment of brain disorders.^2,17^ Despite MTAs being considered a relatively old class of anticancer drugs, some of which are perceived as no longer “trendy”, new therapeutic approaches based on MTAs continue to be proposed for cancer treatment.^18^ However, due to the development of drug resistance and significant side effects associated with these drugs, there is a constant need for more efficient and specific compounds that target tubulin polymerization. Numerous efforts have been made to develop MTA screening methods. In 2016, the team of Klassen et al. developed an assay of antitubulin drugs screening based on catch-and-release electrospray ionization mass spectrometry.^19^ They concluded that the developed assay could be applied for anticancer drug screening and for ranking the affinities of compounds to tubulin. Still, they did not apply this new assay to “real” approved chemical libraries. Moreover, the affinities of compounds are not always directly correlated with the anticancer activity of the molecules. While at least six distinct binding sites for tubulin inhibitors are currently known, each influencing tubulin polymerization in different ways, recent virtual screening studies have expanded the number of potential binding sites to 27.^20^ To validate the virtual screening findings, the development of the functional MTA test is still needed. This encouraged the team of Stefano Di Fiore to develop a SNAP-tag-based screening assay for the analysis of MT dynamics and cell cycle progression.^21^ Unfortunately, like the previous assay, it was tested only on a small number of molecules; however, it follows MT functions, making it more appropriate for new MTA screening. The main disadvantage of the proposed assay is that it follows the impact of tested compounds in the cells wherein it is very difficult to separate the direct impact of the molecules on tubulin polymerization from indirect perturbation of the cellular cytoskeleton through molecules binding to some other targets that perturb cell cycle and thus impact the cytoskeleton. Finally, to the best of our knowledge, until now, there has been no functional high-throughput assay for MTA screening applied to diverse chemical libraries except in silico virtual screenings^22–25^ sometimes followed by further in vitro validation.^22^

The new nanoDSF screening assay introduced in this study not only overcomes the limitations of previous methods but also introduces additional advantages that are crucial for accurately determining the mode of action of compounds. First, nanoDSF serves as an in vitro functional assay within a simplified environment, enabling the ranking of compounds by their effect on tubulin polymerization, as indicated by shifts in *T*_poly_. Second, nanoDSF incorporates several internal controls that enhance the assay’s reliability. The initial fluorescence measurement confirms the correct tubulin concentration, essential since *T*_poly_ is concentration dependent. Additionally, this assay enables the determination of tubulin’s *T*_m_, thereby not only calculating Δ*T*_m_ for each compound but also ensuring the proper folding state of tubulin at each run. This feature is particularly vital for automated screenings, where maintaining the stability of such “fragile” proteins as tubulin over extended periods in plates is a key concern. Furthermore, by employing varying concentrations of compounds, it is feasible to ascertain the apparent association constants of hits with tubulin based on both the fluorescence signal at a fixed temperature and the denaturation temperature shift. Unlike previous MTA screening assays, we validated our method on a library of 1520 approved compounds and successfully identified every known MTA in that set— Colchicine, Docetaxel, Fenbendazole, Mebendazole, Paclitaxel, Podophyllotoxin, Albendazole, Griseofulvin, Nocodazole, Oxibendazole, Oxfendazole, Parbendazole, and Triclabendazole.

### Compounds with Highest Microtubule Targeting Agent Activity

Through a novel nanoDSF screening assay, we identified approximately 95 compounds with MTA activity within the PCL. Among these, 20 compounds fully inhibited temperature-induced tubulin polymerization under the experimental conditions (Table 1). Some achieved this by directly inhibiting polymerization, while others promoted polymerization at lower temperatures. Notably, recognized MTAs such as Paclitaxel, Docetaxel, Podophyllotoxin, Colchicine, Fenbendazole, and Mebendazole were among those that prevented temperature-induced polymerization entirely. Additionally, our study confirmed that artificial estrogens like Hexestrol and Dienestrol, as well as the antiseptic Thiomersal, also impacted tubulin polymerization, consistent with previous reports of their direct effects on tubulin.^26,27^

Over half of the strong hits were identified as MTAs for the first time through this screening. Among these, Auranofin (AUF), Ebselen (EBS), and Riboflavin (RBF) had previously been shown to affect the cytoskeleton despite the absence of direct evidence for tubulin binding. Thus, AUF, an antirheumatic gold complex, inhibits neutrophil activation by markedly reducing the number of centriole-associated MTs and obstructing phagocytosis in human polymorphonuclear leukocytes, likely through a mechanism that involves MT dysregulation.^28,29^ AUF is increasingly recognized as a potential anticancer agent; by serving as an inhibitor of both thioredoxin reductase and proteasome, it induces oxidative stress and triggers apoptosis in models of NSCLC.^30^ EBS, an organoselenium compound that mimics glutathione peroxidase, has been explored as a neuroprotectant in ischemia and conditions linked to oxidative stress.^31^ Research demonstrates its ability to destabilize MTs in skin melanocytes and inhibit tumor growth through the suppression of 6-phosphogluconate dehydrogenase activity.^31–33^ Deficiencies in RBF (vitamin B2) transport are linked to disturbances in MT dynamics; however, these disruptions can be alleviated through the administration of RBF in cellular models.^34^ Furthermore, combining RBF with chemotherapy has been suggested as a strategy to reduce side effects and enhance therapeutic outcomes.^35^

Eight of the identified strong hits were previously unrecognized in their association with tubulin, marking them as novel discoveries. Notably, a family of benzofurans (Benzbromarone (BZB), Benzarone (BZ), and Benziodarone (BZI) Figure 3I) has been demonstrated to directly and effectively modulate tubulin polymerization, aligning with previous findings that BZ inhibits tumor growth in vitro.^36^ Intriguingly, BZ and BZB both were used in the treatment of gout akin to CLH—a well-known MTA. While BZB is believed to act through uric acid reuptake, CLH disrupts inflammasome assembly at the cytoskeletal level.^37,38^ The revelation that benzofurans may also interact with MTs introduces an additional dimension to our understanding of their anticancer and anti-inflammatory properties. Among the novel MTAs identified is APT, a GPCR inhibitor is extensively used in chemotherapy to prevent common side effects like nausea. Remarkably, APT is also attributed with the antitumor properties of its own.^39,40^ BTN and HCP (Figure 3L) are fungicides from a class of bridged diphenyl compounds with cytotoxic and antiproliferative action in cancer cell lines.^41,42^ Our findings reveal that several dihydropyridines, approved for managing angina, also interfere with MT assembly. Notably, Nifedipine (NFD) and Nisoldipine (NSD) (Figure 3G) demonstrated the most significant impact on tubulin. While calcium channel blockers like NFD have been previously noted to enhance the sensitivity of drug-resistant cancer cell lines to PTX,^43^ our research marks the first instance of identifying these medications as MTAs.

Collectively, the strong hits identified in this study present compelling cases for drug repurposing, echoing the findings of prior research. Specifically, compounds such as AUF, EBS, APT, BZ, BZB, and HCP exhibit antitumor activity in vitro.^33,36,40,42,44,45^ NFD shows potential in reversing drug resistance in cancer cells,^43^ while RBF and BTN enhance the effectiveness of existing anticancer drugs.^35,41^ Additionally, BZI and NFD represent modifications of molecules with established antineoplastic properties, further underscoring their potential for repurposing in cancer therapy.

### Structure–Activity Relationship

#### Carbendazim and Benzofuran Clusters

Carbendazim derivatives were characterized (Table S2) from a significant cluster of compounds with varied MTA activity, ranging from the complete inhibition of tubulin polymerization seen in the presence of MBZ and FBZ to a spectrum of high, medium, and low inhibitory effects observed for Albendazole (ABZ, 6.4°C), Nocodazole (NCZ, 6.0°C), Oxfendazole (OFZ, 3.8°C), Methiazole (MTZ, 2.7°C), Parbendazole (PBZ, 1.8°C), Flubendazole (FLU, 1.4°C), and Oxibendazole (OBZ, 1.3°C) (Figure 3D). While most of these derivatives are utilized as broad-spectrum anthelmintic agents targeting the colchicine site on tubulin, PBZ is employed as an antifungal drug, and only NCZ is used in oncology. However, most exhibit anticancer potential to varying degrees. Specifically, MBZ and ABZ have been highlighted as promising anticancer agents,^46,47^ FBZ has shown moderate antineoplastic activity,^48^ OFZ has been found to inhibit cell growth in NSCLC,^49^ MTZ enhances the efficacy of gemcitabine in pancreatic cancer,^50^ and FBZ has a putative action against triple-negative breast cancer.^51^ Even OBZ, with the lowest inhibitory effect on tubulin polymerization among the MBZ derivatives, has been reported to significantly impede the growth of androgen-independent tumors.^52^ Carbendazim itself, along with some of its derivatives,^53^ are systemic broad-spectrum fungicides that also target tubulin.^54^ The most potent inhibitors of tubulin polymerization among its derivatives, MBZ and FBZ, feature a benzene ring attached at the 11th position of the carbendazim structure, connected through a sulfur atom or a carbonyl group. Further analysis reveals that the inhibitory effect on tubulin polymerization, as indicated by changes in Δ*T*_poly_ for OBZ, PBZ, and ABZ, significantly improves with the substitution of carbon atoms at the first position of the aliphatic chain with sulfur and, to a lesser extent, decreases with substitution by oxygen.

We also identified a compact cluster of three benzofuran derivatives: Benzarone (BZ), Benzbromarone (BZB), and Benziodarone (BZI) (Figure 3I, Table S2). All three compounds exhibited complete inhibition of tubulin polymerization. Considering that modification of the phenol group with bromine and iodine does not diminish the inhibitory properties of BZ, it suggests that the benzofuran moiety may play a pivotal role in tubulin polymerization inhibition. Supporting this notion, TCZ, featuring a Benzothiophene group—a structure analogous to benzofuran with the oxygen atom replaced by sulfur—exhibits the most pronounced effect on tubulin polymerization within the MCZ cluster (Figure 3H). This hypothesis is in line with published data on the impact of benzofuran in its derivatives on tubulin polymerization.^55,56^ While there has been no previous report of these compounds exhibiting MTA activity, BZ has been documented to inhibit the growth of colorectal cancer cells both in vitro and in vivo.^36^ Although the mechanism of BZ’s action was suggested to involve its primary target EYA3 and the inhibition of the EYA3-SIX5-p300 complex, our results suggest the possibility of a direct effect on MTs as well. This is supported by observations of BZ leading to a dose-dependent decrease in cell proliferation and invasion,^36^ processes fundamentally reliant on MT dynamics. Additionally, BZB has been pinpointed as a potential candidate for drug repositioning in the treatment of LUAD through AI-driven analysis of gene dysregulation.^44^ Our findings lend robust support to these insights, suggesting a potential molecular mechanism behind BZB’s anticancer efficacy.

#### Tricyclic Molecules Clusters

Several derivatives of PTZ have been identified to exhibit notable MTA activity (Figure 3, Table S1). While PTZ itself induces a modest shift in tubulin polymerization temperature (Δ*T*_poly_) by 1 °C, its derivatives—Thiethylperazine (TEP, 1.0°C), Chlorprothixene (CPX, 1.1°C), Toluidine blue (TB, 1.1°C), Perphenazine (PPZ, 1.5°C), Methylene Blue (MB, 1.7°C), Chlorpromazine (CPZ, 1.7°C), Trifluoperazine (TFP, 2.7°C), Flupentixol (FPX, 3.5°C), Fluphenazine (FPh, 3.7°C), and Triflupromazine (TFZ, 6.4°C)—show progressively stronger inhibitory effects (Figure 3E). These are antipsychotics drugs (except TB and MB) often used in schizophrenia patients, and there is epidemiological evidence that has linked lower cancer incidence in schizophrenia patients to long-term medication, highlighting the anticancer potential of antipsychotics.^57^ Notably, only TFP and CPZ have been previously recognized for their ability to inhibit MT assembly,^58,59^ yet all these derivatives have been either demonstrated or hypothesized to possess anticancer properties, despite their primary classification as CNS therapeutics targeting dopamine receptors. For instance, PPZ has been highlighted as a potential antitumor agent,^60^ CPZ in oral cancer treatment,^61^ TFP in suppressing colorectal cancer cell models,^62^ FPX as a potential lung cancer treatment,^63^ FPh in enhancing cancer cell sensitivity to Halaven,^64^ and TFZ has been identified as a selective modulator affecting the breast cancer cell cycle.^65^ While various mechanisms for the anticancer activity of these drugs have been proposed, our results suggest a direct, shared MTA mechanism among these structurally related molecules.

Additionally, this MTA mechanism might offer an alternative explanation for the pleiotropic effects observed with PTZ derivatives against Gram-negative bacterial persister cells^66^ and their antitubercular activity.^67,68^ Within the screened PTZ derivative family, there are three pairs of molecules in which the substitution of a –Cl group with a –CF_3_ group at the second position (Figure 3D) markedly enhanced their inhibitory effects on tubulin polymerization. Specifically, for the CPZ and TFZ pair, the Δ*T*_poly_ escalated from 1.7 to 6.4 °C; for PPZ and FPh, it increased from 1.5 to 3.7 °C; and for prochlorperazine and TFP, it rose from 0.5 to 2.7 °C (Figure 3D). The critical contribution of the –CF_3_ group to inhibiting tubulin polymerization is also underscored by a 1 °C higher Δ*T*_poly_ observed for 2-(trifluoromethyl) PTZ compared to PTZ.

A similar enhancement in tubulin polymerization inhibition resulting from the substitution of a –Cl group with a –CF_3_ group is observed in another pair of molecules, derivatives of thioxanthene. These differ from PTZ derivatives only by replacement of a nitrogen atom with carbon in the central ring (Figure 3D). Zuclopenthixol (ZPX), bearing a –Cl group at the second position, shows no significant effect on tubulin polymerization, whereas FPX, featuring a –CF_3_ group, induces a 3.5 °C shift in *T*_poly_.

This study also uncovered a group of MTAs among Dibenzosuberone (DBS) derivatives (Figure 3F), traditionally recognized as tricyclic antidepressants: Nortriptyline (NTP), Loratadine (LTD), Protriptyline (PTP), Norcyclobenzaprine (nCBP), Opipramol (OPP), Clomipramine (CMP), and Asenapine (ANP). These compounds exhibit a modest effect on tubulin polymerization, with a Δ*T*_poly_ of approximately 1 °C. While none of these were previously recognized for influencing tubulin polymerization, certain members have been noted for their anticancer activities against prostate^69,70^ or glioblastoma cell lines.^71^ Additionally, CMP has been reported to augment the cytotoxicity induced by vinorelbine in human neuroblastoma cancer cells.^72^ The antitubulin properties of PTZ, Thioxanthene, and DBS derivatives, which are utilized in CNS treatments and thereby capable of crossing the blood–brain barrier, position them as promising candidates for repositioning in the treatment of brain tumors.

## Conclusions

In summary, we have developed a novel nanoDSF assay for screening MT-targeting agents, compounds widely used in anticancer, antifungal, and antibacterial therapies. Unlike previous assays, our method evaluates both compound binding to tubulin and its impact on tubulin polymerization. We validated this assay using the PCL, comprising 1520 approved compounds, successfully identifying all known MTAs as hits. This approach also uncovered new antitubulin drugs among compounds previously associated with cancer cell proliferation inhibition or cytotoxicity, reaffirming tubulin as a critical target for anticancer drug development. Our findings not only pave the way for the drug repositioning of newly identified MTAs and streamline the search for novel scaffolds within large chemical libraries but also facilitate the exploration of structure–activity relationships, contributing to more efficient rational drug discovery. We hope to inspire renewed interest in the discovery of anticancer compounds within the MTA class.

## Experimental Section

### Materials

Human glioblastoma cells were obtained from ATCC (Gaithersburg, MD, USA). Compounds used in the cytotoxicity assay were from PCL, MedChemTronica (Bergkällavägen, Sweden), or Sigma (St Louis, MO, USA).

### Tubulin Purification

Tubulin was purified from lamb brains by ammonium sulfate fractionation and ion-exchange chromatography and stored in liquid nitrogen as described.^73^ Tubulin concentration was determined at 275 nm with an extinction coefficient of 109,000 M^–1^ cm^–1^ in 6 M guanidine hydrochloride.

### Turbidimetry and Differential Scanning Fluorimetry Assays

Aliquots of tubulin were passed through a larger (1 × 10 cm) gravity column of Sephadex G25 equilibrated with 20 mM sodium phosphate buffer, 1 mM EGTA, 10 mM MgCl_2_, 3.4 M glycerol, and 0.1 mM GTP, pH 6.5 (PEMGT buffer) or 20 mM Tris, 1 mM MgCl_2_, 0.1 mM GTP, pH 6.5 for nonpolymerizing conditions. For MTA binding assays, VBL, MBZ, and TXL were used at concentrations up to 100 μM, with tubulin at 15 or 10 μM, respectively. Each capillary was loaded with 10 μL of the sample. Fluorescence and turbidimetry measurements were performed on a nanoDSF Prometheus NT.Plex system equipped with backscattering optics from 15 to 80 °C, using 10% excitation power and a temperature ramp of 1 K/min.

### Nano-Differential Scanning Fluorimetry Screening

For nanoDSF screening of 1520 FDA-approved compounds, 50 nL aliquots in 100% DMSO were dispensed into 384-well microplates and stored at −80 °C. Prior to nanoDSF measurements, 10 μL of 10 μM tubulin in PEMGT buffer was added to each well in a 24-well lane and mixed thoroughly by pipetting. The final compound concentration was approximately 50 μM in 0.5% DMSO. Plates were briefly centrifuged to eliminate air bubbles, and the samples were transferred to standard DSF-grade capillaries mounted on a 24-capillary rack. All measurements were performed on a nanoDSF Prometheus NT.Plex instrument from 20 to 75 °C, with 10% excitation power and a heating rate of 1 K/min.

### Isothermal Titration Calorimetry

Binding of MTA to tubulin was probed using a MicroCal iTC200 instrument (MicroCal, Northampton, MA, USA, now part of Malvern Instruments Ltd., Malvern, UK) in PEMGT buffer. To prevent tubulin polymerization, the cell temperature was maintained at 10 °C. Tubulin was loaded into the calorimetric cell at a concentration of 20 μM, and MTA was titrated with a syringe at 250 μM.

### Transmission Electron Microscopy

Samples were adsorbed onto 200 mesh Formvar carbon-coated copper grids, stained with 2% (w/v) uranyl acetate, and blotted to dryness. Grids were observed using a JEOL JEM-1220 transmission electron microscope operated at 80 kV. Magnifications used range from 60,000× to 120,000×. To ensure that MTs do not disassemble during adsorption, this step was performed in a thermostated room at 37 °C. The same step was performed for grids at 4 and 80 °C.

### Cell Culture, Cytotoxicity, and Proliferation Assays

Glioblastoma cell culture routines, viability, and proliferation assays were performed as previously described.^74^ U87MG cells were maintained in complete MEM media supplemented with 10% FBS and 2 mM l-glutamine (Invitrogen, Paris, France). For cytotoxicity assays, cells were counted and plated in 96-well flat-bottom plates (50,000 cells/mL, 5000 cells per well). After 24 h, the cells were treated with increasing concentrations of the MTAs (0, 1, 5, 10, and 20–40 μM) in a vehicle solution, containing 0.05% DMSO. All concentrations were done in triplicates. The surviving cells were quantified after 72 h by the tetrazolium bromide MTT-assay, according to the manufacturer’s instructions. After cell lysis, the optical density was measured at 600 nm using a Multiskan MS Thermo plate reader (LabSystems, Waltham, MA, USA). Cell viability was expressed as a percentage of survival, using cells treated with the vehicle solution as 100%, and the IC_50_ values were calculated by using the Chou and Talalay linearization method.^75^

### Funnel Metadynamics

We employed three freely available modern force fields, including Amber19sb. All ligands were parametrized using acpype, with atom point charges derived from ab initio 6-31G calculations utilizing psiresp.^76^ Additional parameters were assigned according to the GAFF2 force field. GDP was parametrized in the same manner. The structure of tubulin alpha was modeled based on the coordinates from PDB ID 4o2b.^77^ Protonation states of residues were predicted using PROPKA and manually verified.^78^ The system was then solvated in a triclinic box with periodic boundary conditions by using TIP3P water molecules. To neutralize the system and achieve an ionic strength of 0.15 M, Na^+^ and Cl^–^ ions were added. Energy minimization was performed using 5000 steps of the steepest descent method. The equilibration phase comprised seven steps. Initially, a 100 ps NVT simulation was conducted, applying positional restraints of 1000 kJ/(mol nm^2^) to the heavy atoms. Temperature coupling was maintained with a velocity rescale thermostat.^79^ This was followed by five rounds of NPT equilibration, each lasting 100 ps, during which restraint strength was gradually reduced: 1000, 500, 200, 100, and 10 kJ/(mol nm^2^). Pressure coupling was achieved using a stochastic barostat.^80^

The funnel metadynamics setup was modeled after the approach described by Raniolo and Limongelli.^81^ A metadynamic potential of 0.5 kJ/mol was applied every 500 steps. Two collective variables were employed: first, the distance between the COM of CLH in its binding site and a reference point is 20 Å away from its position. This variable projected the ligand along the funnel line. Second, the perpendicular distance of the ligand from this line. A correction to the binding free energy was applied to account for the entropic contribution due to the funnel-shaped restraint, following the equation provided in Raniolo and Limongelli.^81^ The correction factor for the cylinder was calculated as 1.59 kcal/mol. Final binding free energies were reported with error estimates, following the method of Bhakat and Söderhjelm,^82^ using a statistical analysis window of 1000 ns.

## Supplementary Material



## Data Availability

All data available upon request.

## Notes

PTZ, thioxanthene, and dibenzosuberane clusters; carbendazim and coumarone clusters; miconazole cluster; NFD cluster; stilbenoids clusters; steroids cluster; diphenyls clusters; and nonclustered hits (PDF)

## Abbreviations

ITC: isothermal titration calorimetry
MAP: microtubule-associated protein
MT: microtubules
MTA: microtubule targeting agents
nanoDSF: nano-differential scanning fluorimetry
PCL: Prestwick Chemical Library
TEM: transmission electron microscopy
TSA: thermal shift assay
