# Supplementary material for: NanoDSF Screening for Anti-tubulin Agents Uncovers New Structure–Activity Insights
Source: J Med Chem. 2025 Aug 15;68(16):17485–98. doi: 10.1021/acs.jmedchem.5c01008 (PMC12406199; doi:10.1021/acs.jmedchem.5c01008)
Supplement: Supplementary file 1 [file jm5c01008_si_001.pdf]

Supplementary information
NanoDSF Screening for Anti-Tubulin Agents Uncovers New Structure–Activity Insights

Viktoriia Baksheeva[a], Romain La Rocca[a], Diane Allegro[a], Carine Derviaux[b], Eddy Pasquier[b], Philippe Roche[b], Xavier Morelli[b], François Devred[a,c], Andrey V Golovin[d], Philipp O Tsvetkov* [a,c]

[a] Aix-Marseille Univ, CNRS, INP, Inst Neurophysiopathol, 13005 Marseille, France;

[b] Aix-Marseille Univ, CNRS, INSERM, Institut Paoli Calmettes, CRCM, Centre de Recherche en Cancérologie de Marseille, 13009 Marseille, France;

[c] Aix-Marseille Univ, PINT, Plateforme Interactions moléculaires Timone, Faculté des Sciences Médicales et Paramédicales, 13005 Marseille, France.

[d] Faculty of Bioengineering and Bioinformatics, Belozersky Institute of Physico-Chemical Biology, Lomonosov Moscow State University, 119991 Moscow, Russia.

* corresponding author: philipp.tsvetkov@univ-amu.fr

Contents of SI

Table S1. Phenothiazine, Thioxanthene, and Dibenzosuberane clusters

Name	Code	ΔT_{poly},°C	ΔT_m,°C	IC_{50}, µM
**Phenothiazine group**				
Phenothiazine*	PTZ	1.0	-0.5	60.2±9.2
Thiethylperazine*	TEP	1.0	-0.6	8.4±0.2
Perphenazine	PPZ	1.5	-0.6	9.8±0.4
Chlorpromazine	CPZ	1.7	-0.6	4.5±0.5
Trifluoperazine	TFP	2.7	-0.5	17.3±3.2
Fluphenazine	FPh	3.7	-0.4	7.5±0.5
Triflupromazine	TFZ	6.4	-0.3	6.5±0.6
**Thioxanthene group**				
Chlorprothixene	CPX	1.1	-0.3	27.6±2.2
Flupentixol	FPX	3.5	-0.3	9.8±1.8
**Dibenzosuberane group**				
Norcyclobenzaprine*	nCBP	0.9	-0.3	15.2±0.8
Loratadin*	LTD	0.9	0.0	25.0±3.8
Nortriptyline*	NTP	0.9	-0.4	20.5±1.5
Opipramol*	OPP	0.9	-0.2	n/d
Protriptyline hydrochloride	PTP	1.1	-0.1	17.7±1.5
Asenapine	ANP	1.1	-0.2	22.5±4.0
Clomipramine	CMP	1.6	-0.3	19.6±0.6
**Other tricyclic compounds**				
Tolonium (Toluidine Blue O)	TBO	1.1	-0.4	6.3±0.3
Methylene Blue	MB	1.7	-0.4	64.2±9.9
Riboflavin	RBF	n/a	-0.3	≫60

n/a - not applicable; n/d - not determined, * - not considered as hits, included for SAR analysis.

Table S2. Carbendazim and Coumarone clusters

Name	Code	ΔT_{poly},°C	ΔT_m,°C	IC_{50}, μM
**Carbendazim group**				
Fenbendazole	FBZ	n/a	-0.2	0.2±0.0
Mebendazole	MBZ	n/a	0.3	≪0.1
Astemizole	ASZ	1.1	-0.1	1.7±0.1
Oxibendazol	OBZ	1.3	-0.2	1.8±0.3
Flubendazol	FLU	1.4	-0.3	0.6±0.0
Parbendazole	PBZ	1.8	-0.2	≪0.1
Triclabendazole	TCZ	2.2	-0.5	15.1±0.9
Methiazole	MTZ	2.7	-0.2	n/d
Oxfendazole	OFZ	3.8	-0.2	17.0±1.0
Nocodazole	NCZ	6.0	-0.3	<0.1
Albendazole	ABZ	6.4	-0.2	0.3±0.0
**Coumarone group**				
Benzarone	BZ	n/a	0.2	7.4±0.7
Benzbromarone	BZB	n/a	-0.5	62.1±2.8
Benziodarone	BZI	n/a	-0.6	n/d

n/a - not applicable; n/d - not determined.

Table S3. Miconazole cluster

Name	Code	ΔT_{poly},°C	ΔT_m,°C	IC_{50}, μM
Econazole*	ECN	0.9	-0.4	8.0±1.2
Isoconazole	ICZ	1.9	-0.4	33.1±1.8
Miconazole	MCZ	2.3	-0.5	12.9±0.7
Sertaconazole nitrate	STZ	3.8	-0.6	10.7±1.3
Tioconazole	TOZ	4.3	-0.5	18.3±1.4

* - not considered as hits, included for SAR analysis.

Table S4. Nifedipine cluster

Name	Code	ΔT_{poly},°C	ΔT_m,°C	IC_{50}, μM
Nifedipine	NFD	n/a	-0.7	40.4±6.6
Nisoldipine	NSD	n/a	-0.7	25.3±1.6
Felodipine	FDP	1.2	-0.8	13.7±4.0
Nitrendipine	NTD	1.4	-0.3	36.8±3.3

n/a - not applicable.

Table S5. Stilbenoids clusters

Name	Code	ΔT$_{poly}$,°C	ΔT$_m$,°C	IC$_{50}$, μM
**Stilbestrol group**				
Dienestrol	DE	n/a	-0.5	23.6±1.0
Hexestrol	HXS	n/a	-0.4	20.2±0.5
Diethylstilbestrol	DES	2.7	-0.3	n/d
**Tamoxifen group**				
Toremifene	TMF	1.9	-0.1	13.3±1.9
(Z,E) Clomiphene citrate	CMX	2.6	-0.7	5.0±1.0
Tamoxifen citrate	TMX	7.0	-0.6	n/d

n/a - not applicable; n/d - not determined.

Table S6. Steroids cluster

Name	Code	ΔT$_{poly}$,°C	ΔT$_m$,°C	IC$_{50}$, μM
Estramustine*	EM	0.9	-0.3	≫60
Methandrostenolone	MAS	1.4	0.1	58.7±5.1
17β-estradiol	ED	1.5	-0.2	30.7±1.9
Norgestimate	NGS	2.8	-0.5	≫60
Ethinylestradiol	EES	3.9	-0.3	15.7±0.8
Testosterone propionate	TP	-1.1	0.1	n/d
Deflazacort	DFC	−1.3	0.0	≫60
Gestodene	GD	−2.8	-0.3	n/d

n/d - not determined. * - not considered as hits, included for SAR analysis.

Table S7. Diphenyls clusters

Name	Code	ΔT$_{poly}$,°C	ΔT$_m$,°C	IC$_{50}$, μM
**Benzophenone group**				
Oxybenzone	OXY	1.5	-0.2	14.4±7.0
Dioxybenzone	DOB	1.8	-0.2	≫60
**Diphenylmethane group**				
Hexachlorophene	HCP	n/a	0.0	5.1±0.7
Lidoflazine	LFZ	1.1	-0.1	n/d
Diclazuril	DCZ	1.7	-0.6	48.9±6.0
Dichlorophen	DCP	2.7	-0.3	7.7±1.2
Mitotane	MTT	2.7	-0.1	23.7±2.7
**Diphenyl ethers group**				
Triclosan	TCL	2.9	-0.7	11.6±0.5
Liothyronine	LTY	−2.7	-0.1	≫60
(L)-Thyroxine	TYX	−2.6	-0.2	≫60
**Other diphenyl compounds**				
Bithionol	BTN	n/a	0.2	≫60
Meclofenamic acid	MFA	2.9	-0.7	n/d

n/a - not applicable; n/d - not determined.

Table S8. Nonclustered hits

Name	Code	ΔT_{poly},°C	ΔT_m,°C	IC_{50}, μM
**Known MTAs**				
Thiomersal	TMS	n/a	-1.0	2.1±0.1
Colchicine	CLH	n/a	2.2	n/d
Docetaxel	DTX	n/a	7.2	n/d
Paclitaxel	PTX	n/a	5.9	n/d
Podophyllotoxin	PPT	n/a	1.2	n/d
Ethacrynic acid	ECA	1.3	-0.6	≫60
Griseofulvin	GSF	2.0	-0.2	24.4±3.7
Phenolphthalein	PP	3.6	-0.4	≫60
Disulfiram	DFM	−6.5	-0.4	12.0±1.6
**New MTAs with known anti-cancer activity**				
Aprepitant	APT	n/a	-0.2	41.8±31.8
Auranofin	AUF	n/a	-0.6	0.9±0.6
Ebselen	EBS	n/a	0.2	50.9±32.7
Acemetacin	AMT	1.1	-0.3	≫60
Fluoxetine	FX	1.1	-0.2	26.5±4.1
Flurbiprofen axetil	FBA	1.2	0.2	≫60
Troglitazone	TGZ	1.3	-0.7	39.5±1.0
Propofol	PPF	1.4	0.0	26.6±2.5
Mefloquine	MFQ	1.5	-0.5	11.6±1.2
Clotrimazole	CTM	1.6	-0.4	6.1±0.6
Sertindole	STD	2.2	-0.4	6.7±0.2
Efavirenz	EFV	5.6	-0.6	26.7±2.6
Tizanidine	TZN	−1.3	0.1	n/d
Haloprogin	HPG	−1.4	-0.1	n/d
Betamethasone	BMS	−1.4	-0.1	≫60
Mesalamine	MSL	−1.6	0.2	≫60
Artesunate	ASN	−1.6	0.0	24.0±4.1
Formoterol fumarate	FMT	−1.8	0.2	≫60
Nafamostat	NFM	−2.8	-0.1	45.2±6.1
**New MTAs with unknown effect on cancer cells**				
Merbromin	MBR	2.4	-0.5	≫60
Dihydrostreptomycin	DHS	1.3	0.3	≫60
Clorsulon	CLS	−1.1	-0.1	66.8±13.3
Nefiracetam	NFC	−1.2	0.0	≫60
Pramipexole	PPX	−1.2	0.1	n/d
Paroxypropione	POP	−2.5	0.0	n/d
Colistin	CST	−3.0	-0.2	≫60

n/a - not applicable; n/d - not determined.
